# Supplementary material for: Mutations in PCYT2 disrupt etherlipid biosynthesis and cause a complex hereditary spastic paraplegia
Source: Brain. 2019 Oct 22;142(11):3382–97. doi: 10.1093/brain/awz291 (PMC6821184; doi:10.1093/brain/awz291)
Supplement: awz291_Supplementary_Data [file awz291_supplementary_data.zip › awz291-Suppl_data/Supplementary_Data4.zip!/Supplementary information 3 - fibroblast lipidomics.zip!/Fibroblast lipidomics/Controle vs Patient1/Statistical plots and data/Volcano plot_alternative.pdf]

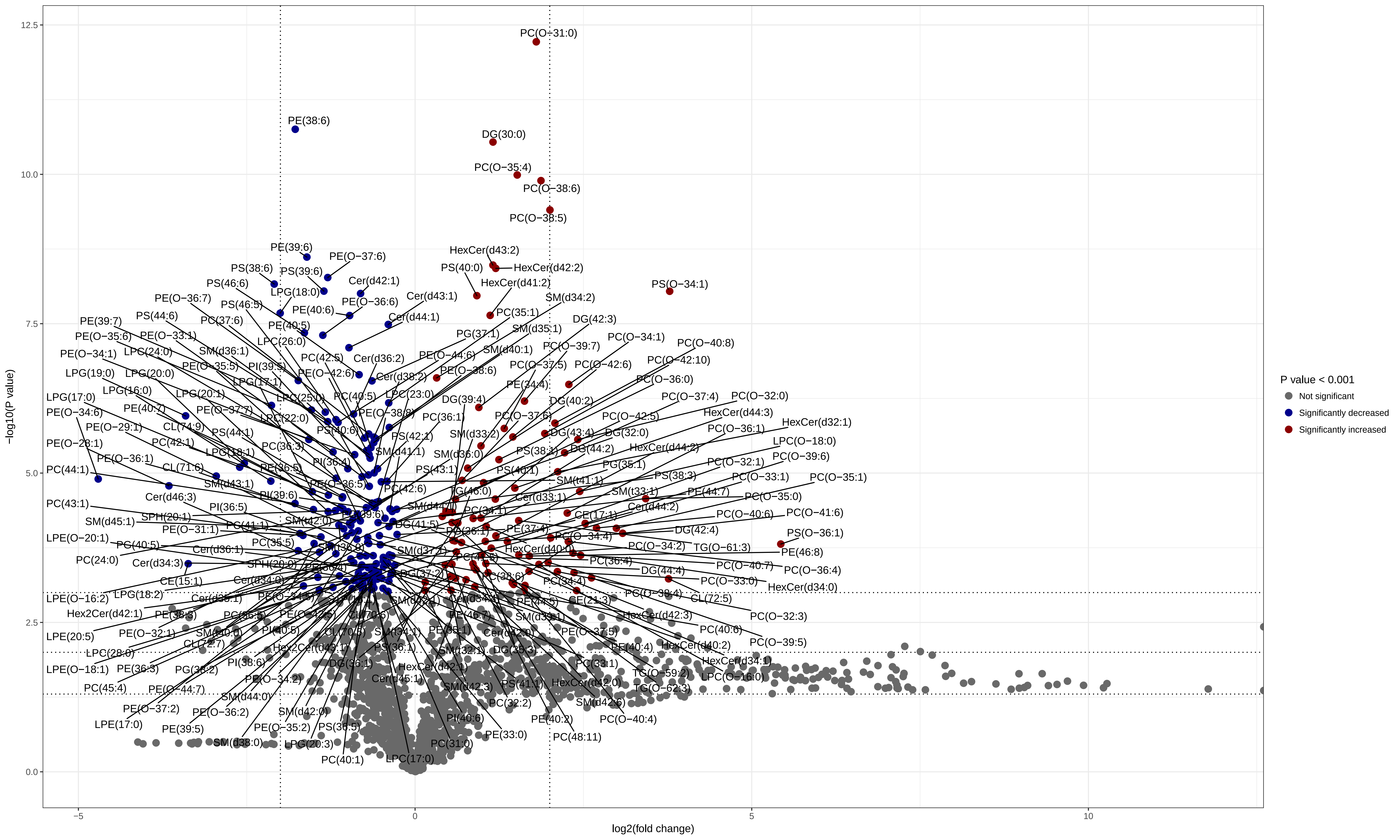

−log10(P value)
log2(fold change)
12.5
10.0
7.5
5.0
2.5
0.0
−5
0
5
10
P value < 0.001
Not significant
Significantly decreased
Significantly increased
PC(O−31:0)
PE(38:6)
DG(30:0)
PC(O−35:4)
PC(O−38:6)
PC(O−38:5)
PE(39:6)
PE(O−37:6)
HexCer(d43:2)
HexCer(d42:2)
PS(40:0)
PS(38:6)
PS(39:6)
Cer(d42:1)
HexCer(d41:2)
PS(O−34:1)
PS(46:6)
LPG(18:0)
Cer(d43:1)
SM(d34:2)
PE(O−36:7)
PS(46:5)
PE(O−36:6)
PE(40:6)
Cer(d44:1)
PC(35:1)
DG(42:3)
PE(39:7)
PS(44:6)
PC(37:6)
PE(40:5)
SM(d35:1)
PE(O−35:6)
PE(O−33:1)
LPC(26:0)
PG(37:1)
PC(O−34:1)
PC(O−40:8)
PC(O−39:7)
PE(O−34:1)
LPC(24:0)
SM(d36:1)
PC(42:5)
Cer(d36:2)
PE(O−44:6)
SM(d40:1)
PC(O−42:10)
PE(O−35:5)
PI(39:5)
PE(O−38:6)
PC(O−37:5)
PC(O−42:6)
LPG(19:0)
LPG(20:0)
LPG(17:1)
PE(O−42:6)
Cer(d38:2)
PC(O−36:0)
PE(34:4)
LPG(16:0)
LPG(20:1)
LPC(23:0)
PC(O−37:4)
PC(O−32:0)
LPG(17:0)
LPC(25:0)
PC(40:5)
DG(39:4)
DG(40:2)
PE(40:7)
PE(O−37:7)
PE(O−38:8)
HexCer(d44:3)
PE(O−34:6)
LPC(22:0)
PC(36:1)
PC(O−37:6)
PC(O−42:5)
HexCer(d32:1)
PE(O−29:1)
CL(74:9)
PS(40:6)
SM(d33:2)
DG(43:4)
DG(32:0)
PC(O−36:1)
PS(44:1)
PS(42:1)
LPC(O−18:0)
PE(O−28:1)
PC(42:1)
PC(36:3)
SM(d41:1)
PS(38:1)
DG(44:2)
HexCer(d44:2)
PC(O−39:6)
LPG(18:1)
SM(d36:0)
PE(O−36:1)
PI(36:4)
PG(35:1)
PC(O−32:1)
PC(44:1)
CL(71:6)
PE(36:5)
PS(43:1)
PS(40:1)
PS(38:3)
PC(O−33:1)
PC(O−35:1)
SM(d43:1)
PE(O−36:5)
SM(t41:1)
PC(42:6)
TG(46:0)
SM(t33:1)
PE(44:7)
Cer(d46:3)
PI(39:6)
Cer(d33:1)
PC(O−35:0)
PC(43:1)
PI(36:5)
SM(d44:0)
Cer(d44:2)
SPH(20:1)
PE(39:6)
PC(34:1)
CE(17:1)
PC(O−40:6)
PC(O−41:6)
SM(d45:1)
SM(d42:0)
PE(O−31:1)
PC(41:1)
DG(41:5)
PE(37:4)
DG(42:4)
PC(36:1)
PS(O−36:1)
LPE(O−20:1)
PC(O−34:4)
PC(35:5)
PC(40:5)
SM(t36:0)
SM(d37:1)
HexCer(d40:0)
PC(O−34:2)
TG(O−61:3)
Cer(d36:1)
PE(46:8)
PC(24:0)
PG(41:5)
PC(36:4)
Cer(d34:3)
SPH(20:0)
PE(36:4)
PC(O−40:7)
PC(O−36:4)
DG(44:4)
PG(37:2)
CE(15:1)
Cer(d34:0)
PC(38:6)
PC(34:4)
PC(O−33:0)
HexCer(d34:0)
LPG(18:2)
PE(O−34:5)
PC(O−38:4)
LPE(O−16:2)
Cer(d35:1)
SM(d41:1)
SM(d42:1)
Cer(d34:4)
PE(44:5)
CE(21:3)
CL(72:5)
Hex2Cer(d42:1)
PE(38:3)
PC(36:5)
PE(O−42:5)
CL(70:6)
PE(46:7)
SM(d39:1)
HexCer(d42:3)
PC(O−32:3)
LPE(20:5)
PE(O−32:1)
SM(d40:0)
PI(40:5)
CL(70:5)
SM(34:1)
PE(35:1)
Cer(d42:0)
PE(O−37:5)
PC(40:6)
CL(72:7)
Hex2Cer(d43:1)
PS(36:1)
PE(40:4)
HexCer(d40:2)
PC(O−39:5)
LPC(28:0)
SM(d32:1)
DG(35:3)
PI(38:6)
DG(36:1)
PC(33:1)
HexCer(d34:1)
LPE(O−18:1)
PE(36:3)
PG(38:2)
HexCer(d42:1)
TG(O−59:2)
LPC(O−16:0)
PE(O−34:2)
Cer(d45:1)
PS(41:1)
HexCer(d42:0)
PC(45:4)
PE(O−44:7)
SM(d42:3)
TG(O−62:3)
SM(d44:0)
SM(d42:6)
PC(32:2)
PE(O−37:2)
PE(O−36:2)
SM(d42:0)
PE(40:2)
PC(O−40:4)
LPE(17:0)
PI(40:6)
PE(39:5)
PE(O−35:2)
PS(36:5)
PE(33:0)
PC(48:11)
SM(d38:0)
LPC(20:3)
PC(31:0)
PC(40:1)
LPC(17:0)
